# 

**Published:** 1897-09-04

**Authors:** 


					" THE HOSPITAL. ABSOLUTELY PURE, therefore BEST. Sept. 4ih, 1897. ?
1 CADBURY'S ?fw CADBURY'S 3
^Ksas /SErfi a
cocoa ?-m linl m cocoa , ,,Qp -
" The standard of highest purity at present JJL <2?2k JML1 NQ ALifALIES USED. L "H#/,
attainable."? Lanr.tU '
No. 571. Vol. XXII. Sattoeat,
Sept. 4th, 1897.
'SubSseoPp0?8GermS,J PRICE TWOPENCE.

				

